# Template Assisted Generation of Chiral Luminescence in Organic Fluorophores

**DOI:** 10.3389/fchem.2020.557650

**Published:** 2021-01-15

**Authors:** Sonia Maniappan, Ashok Badrinarayan Jadhav, Jatish Kumar

**Affiliations:** Department of Chemistry, Indian Institute of Science Education and Research (IISER) Tirupati, Tirupati, India

**Keywords:** circularly polarized luminescence, chiral templates, nematic liquid crystals, chiral gelators, molecular aggregates, biomolecules

## Abstract

Development of efficient ways of fabricating chiral light emitting materials is an active area of research due to the vast potential offered by these materials in the field of optoelectronic devices, data storage, and asymmetric synthesis. Among the various methods employed, template assisted generation of chiral luminescence is gaining enormous attention due to its simplicity, applicability over a wide range of fluorescent molecules/dyes, and the display of high anisotropic values.

## Introduction

The term polarization of light is enclosed with advanced optical phenomenon which provides vital information on the notable optical features like surface shape, material content etc. Circular polarization is considered to be of significance among the other forms of polarized light as it has gained immense popularity due its inevitable applications in 3D displays, optical sensors, bio-imaging, electroluminescent devices and asymmetric synthesis (Schadt, 1997; Maeda et al., 2011). Single photon exists in two circular polarization states due to their quantum properties (uncharged, massless bosons with quantized spin of ±1 ħ) (Andrews, 2011; Sánchez-Carnerero et al., 2015). When the path followed by the electric and magnetic field vectors is in clockwise helix as the wave propagates to the observer, then it is termed as right circular polarized state whereas when the path followed is in anti-clockwise direction it is said to be left circular polarization state. Circularly polarized luminescence (CPL) spectroscopy is based on the differential emission of the left and right circularly polarized light by intrinsically chiral non-racemic luminescent systems, or from the luminophores that are present in chiral environment. Circular dichroism spectroscopy is often employed to study the ground state properties of chiral material whereas CPL measurement gives information on the excited state chiral properties. Hence, CPL can be employed for the observation of certain transitions which cannot be readily visualized in absorption (Nakanishi et al., 2013; Sang et al., 2019).

Materials in an asymmetric environment and exhibiting luminescence properties show CPL activity. The magnitude of CPL is generally quantified using a term known as luminescence dissymmetry factor (*g*_lum_), which is described as the ratio of difference in the intensities of the left (*I*_*L*_) and right (*I*_*R*_) circularly polarized light to the average total luminescence intensity (Zinna and Di Bari, 2015).

$$\begin{array}{l} g_{lum}=2\;\left(\frac{I_{L}-\;I_{R}}{I_{L}+\;I_{R}}\right) \end{array}$$

**Figure 1:**
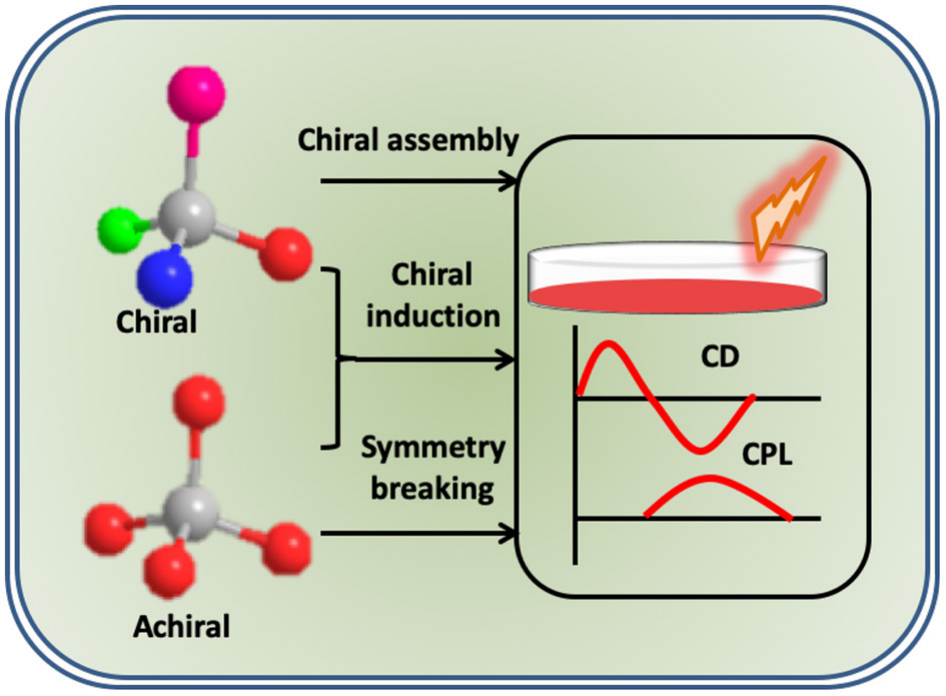
Scheme illustrating the different approaches adopted for the enhancement of optical activity in chiral and achiral molecular systems.

To date, a variety of CPL active molecules and materials have been developed. Chiral lanthanide complexes, transition metal complexes, π-conjugated polymers and small organic molecules, are some examples of systems exhibiting chiral emissive property. Lanthanide complexes, owing to their magnetic dipole allowed transitions, exhibit high *g*_lum_ values; however, narrow and fixed emission bands, and weak luminescence quantum yields are the limiting factors (Zinna and Di Bari, 2015). In contrast, organic chromophores, due to their high luminescence and tunable emission, have attracted vast attention. However, CPL anisotropy is in general weak and in the order of 10^−3^ or less (Sánchez-Carnerero et al., 2015). Various strategies have been adopted for the generation as well as enhancement of CPL activity in these molecular systems. These include techniques like chiral blending, supramolecular assemblies, and template-assisted CPL generation (Maeda et al., 2011; Kumar et al., 2015b). There are comprehensive reviews on the enhancement of CPL activity through supramolecular approaches (Kumar et al., 2015a). Even though, the anisotropy factor could be increased by around an order of magnitude, the values remained low for any technological applications. In contrast, helical templates or chiral host matrices can lead to chiral induction in achiral chromophores through the symmetry breaking imposed by the asymmetric nature of the scaffold (Figure 1). Owing to its large chiral induction effects, these techniques have displayed great potential for the fabrication of CPL active materials with enhanced luminescence dissymmetry (Mei et al., 2015). This review mainly focuses on the generation of CPL active organic luminescent molecules utilizing the template assisted methods and provides an overview about the various templates employed till date.

## Liquid Crystals

Chiral liquid crystals are the most widely used templates for the induction of optical activity in molecules and materials. Liquid crystals are classic soft materials having intermediate phase between solid crystal and isotropic liquid (Kim et al., 2019). They possess long-range orientational order exhibiting interesting thermal, mechanical and optoelectrical properties. Chiral nematic liquid crystals (^*^NLCs) can be obtained (i) directly from cholesteric liquid crystals or (ii) from nematic liquid crystals doped with chiral additives. Owing to their helical order and periodic structure, ^*^NLCs exhibit fascinating optical properties (Li et al., 2018). One of the most interesting features that make these materials attractive is the selective reflection of circularly polarized light passing through them. When the wavelength of incident light is in resonance with the wavelength of selective reflection, circularly polarized light of same handedness as the liquid crystalline matrix is selectively reflected and light with opposite handedness is transmitted. This is in particular important for the generation of high anisotropic factors from ^*^NLCs-based nanosystems (San Jose et al., 2014). The wavelength of the reflected light (λ_0_) depends on the helical pitch (*P*), refractive index of the cholesteric material (*n*) as well as the angle (θ) between the incident light and the cholesteric layers (Fernandes et al., 2019). It can be defined as:

$$\begin{array}{l} \lambda_{0}=nP\sin\theta \end{array}$$

Furthermore, induced chirality can be generated by the helical arrangement of achiral molecules on chiral templates. Thus, liquid crystalline materials doped with achiral molecules exhibit intense chiroptical signals and have great potential for applications in stereoscopic displays and color-image projection.

The idea of generating CPL with high anisotropic factor by using liquid crystalline template was introduced by Pollmann et al. (1976) by embedding fluorescent dye in a ^*^NLC host. Later, experimental and theoretical studies were carried out by Shi et al. (1998) for emission outside the selective reflection band (or the resonance region) of the liquid crystalline material. ^*^NLC films doped with a fluorescent dye displayed CPL with chiral anisotropy factor around 0.8 (Shi et al., 1998). In contrast, for emission inside the resonance region, almost pure circular polarization (*g*_lum_ = ~2) was observed for ^*^NLC films embedded with light-emitting dopants (Chen et al., 1999). Later, a large variety of liquid crystalline matrices and achiral fluorophores were employed for the generation of intense CPL signals. Akagi's group has extensively reported on the research topic. One of their works demonstrated the preparation of a CPL-switchable cell containing a CPL-emitting film and chiral disubstituted liquid-crystalline polyacetylene (di-LCPA) merged with a thermoresponsive N^*^-LC cell. CPL switching and amplification could be achieved by the changes in the phase of thermotropic ^*^NLC cell and the selective transmission of CPL in ^*^NLC (San Jose et al., 2014). Another area in which these materials have been used widely is the polymerization reactions, wherein ^*^NLC function as chiral solvents. Helical network polymers exhibiting CPL activity was synthesized through photo-crosslinking polymerization of functionalized methacrylates in chiral ^*^NLC as asymmetric solvent for polymerization (Park et al., 2015).

The major limitation with systems having high concentration of fluorophores in ^*^NCs is the quenching associated with aggregation. To overcome this, Zhao et al. (2016) used organic luminogens possessing aggregation-induced emission (AIE) property. AIE-LC synthesized by doping tetraphenylethylene-propylphenylethyne (TPE-PPE) in chiral ^*^NLCs showed intense CPL properties (Zhao et al., 2016). Li et al. (2018) in contrary, developed nanocomposites by embedding a chiral AIE luminogen, *R*/*S* BINOL-CN into achiral NLC (N-LC, E7). Considerably high CPL response with a *g*_lum_ value of +0.41 was obtained due to the dipolar interactions between polar cyano groups and π-π interactions between binaphthyl moiety of *R/S*-BINOL-CN and biphenyl/terphenyl groups of the E7 host (Li et al., 2018). Due to their unique properties, ^*^NLCs were also used as scaffolds for the generation of photon upconverted CPL. Triplet–triplet annihilation-based photon upconversion as well as upconverted CPL was observed by dispersing a chiral emitter and a triplet donor into the ^*^NLC (Yang X. et al., 2019).

**Figure 2:**
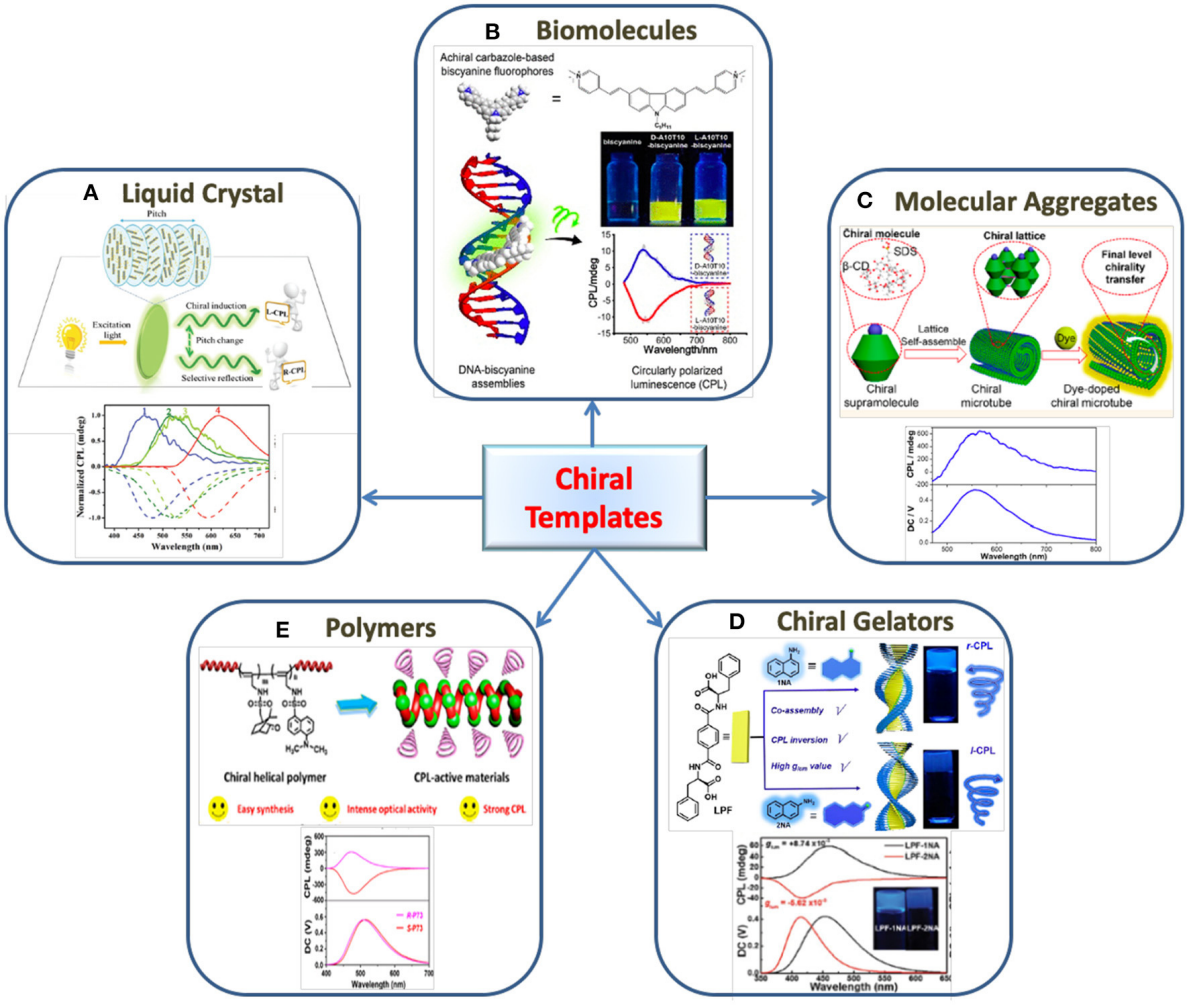
The use of various chiral templates for the generation of CPL in organic fluorophores. **(A)** Liquid crystals: Schematic illustration represents the generation of R-CPL and L-CPL from CNC- fluorescent dyes composite film with respect to helical pitch change and their corresponding CPL spectra. Reproduced with permission from He et al. (2019). Copyright (2019) The Royal Society of Chemistry. **(B)** Biomolecules: Fabrication of CPL material composed of achiral biscyanine and DNA duplex as chiral template. Reproduced with permission from Jiang Q. et al. (2019). Copyright (2019) American Chemical Society. **(C)** Molecular aggregates: Induced CPL generated by blending achiral dyes into helical microtube obtained from self-assembly of β-cyclodextrin (β- CD) and sodium dodecyl sulfate (SDS). Reproduced with permission from Liang et al. (2020). Copyright (2020) American Chemical Society. **(D)** Chiral gelators: scheme illustrates the chirality transfer from a C2-symmteric hydrogelator (LPF) to achiral naphthylamine isomers and the CPL inversion. Reproduced with permission from Yang L. et al. (2019). Copyright (2019) The Royal Society of Chemistry. **(E)** Polymers: CPL obtained from a chiral fluorescent helical mono-substituted polyacetylenes prepared by the copolymerization between a chiral monomer and achiral fluorescent monomer. Reproduced with permission from Zhao et al. (2018). Copyright (2018) American Chemical Society.

Among various ^*^NLC materials, cellulose nanocrystals (CNC) have attracted special attention due to their abundance, stability and robustness. Different approaches such as application of stress, addition of metal salt, change in pH and solvent polarity have been employed to tune the CPL emission of achiral luminogens embedded in CNC templates. In most cases, variations in the pitch of the helix induced by external stimuli were the major cause for the changes in optical properties (Figure 2A). The inversion in the sign of CPL and the tuning of CPL wavelength could be achieved (Zheng et al., 2018; He et al., 2019). Moreover, suitably designed fluorescence resonance energy transfer (FRET) and charge transfer systems have also been used to tailor the CPL emission using ^*^NLC templates (Li et al., 2020; Lin et al., 2020). Thus, it is observed that doping achiral fluorescent dyes with ^*^NLC is emerging as an effortless and efficient way of generating CPL active materials.

## Biomolecules

While substantial progress has been made in the development of CPL active materials with different chiral templates, the development of probes using nucleic acids or related biostructures has remained unexplored to large extend. Biomolecules such as DNA and RNA owing to their well-defined molecular backbone has the advantage of having functional groups placed at a defined space and distance. This enables ordered arrangement of guest molecules at specified locations on the scaffold resulting in a distinct helical organization of the luminophores. The helical assembly gives rise to reproducible chiral luminescence due to the robustness of the template. Nakamura et al. (2016) demonstrated that the pyrene fluorophores when assembled helically like a zipper along the RNA duplex resulted in strong excimer fluorescence as well as CPL. The chiral emission exhibited a dissymmetric factor around 3.5 × 10^−2^ in a dilute aqueous solution (Nakamura et al., 2016). They further extended their investigations to DNA duplexes possessing varying number of pyrenes modified non-nucleosidic linkers wherein CPL emission was observed from the pyrene excimers (Nakamura et al., 2018). In an interesting investigation, Jiang Q. et al. (2019) demonstrated the development of a new class of CPL-active biomaterials using achiral carbazole-based biscyanine fluorophores assembled on chiral DNA scaffold (Figure 2B). Electrostatic attraction drives the binding of achiral cyanine molecules to the minor groove of DNA leading to chirality transfer from the DNA molecules to the cyanine dyes. This resulted in a remarkably high CPL emission from the dye molecules which could be regulated by varying the structure of the template (Jiang Q. et al., 2019). Further, Chen et al. (2019) demonstrated CPL activity in achiral ThT dyes through the interaction with human telomeric G-quadruplex. Statistical analysis of different DNA sequences and structures revealed that right- and left-handed CPL are induced on the parallel and antiparallel G4 structure, respectively (Chen et al., 2019). Hence, the exploration of biocompatible templates for CPL emission is emerging as an attractive area of research due to the vast potential of these materials as biosensing and bioimaging platforms.

## Molecular Aggregates

Molecular self-assembly has been demonstrated as an efficient approach for the enhancement of chiral luminescence, particularly, in chiral organic systems. However, there are few reports in which supramolecular assemblies have been employed as chiral templates to induce optical activity in achiral molecules. In a recent report, Liang et al. (2020) utilized the helical microtubes formed by the self-assembly of β-cyclodextrin-sodium dodecyl sulfate supramolecular complex to induce CPL in achiral dyes loaded in the microtubes (Figure 2C). The systems exhibited chirality at different levels starting from β-CD to β-CD/SDS complex, their self-assembled microtubes and finally induced chirality in achiral dyes blended in the microtubes. The achiral dyes exhibited strong induced CPL with a *g*_lum_ value of 0.1 (Liang et al., 2020). Zhang et al. (2019) synthesized two AIE-active chiral Au(I) complexes which undergo spontaneous hierarchical self-assembly transforming from vesicles to helical fibers. Utilizing the enantiomers as chiral transcription templates, induction of CPL signals from achiral luminogens were successfully achieved with *g*_lum_ values around 10^−3^ (Zhang et al., 2019). Goto et al. (2017) could successfully induce CPL in commercially available achiral dyes using chiral nanofibrillar templates synthesized from L-glutamic acid substituted amphiphilic molecules. The nanosystem exhibited enhanced CPL activity with a *g*_lum_ value as high as 0.1 in dilute solution (Goto et al., 2017). The advantages with such self-assembled molecular templates is that they provide wide range of possibility in the choice of molecular building blocks thereby enabling the development of CPL active materials with tailored optical properties.

## Chiral Gelators

Supramolecular organogels comprising of hydrogels and low-molecular-weight organogels (LMWGs) are an important class of soft materials that have shown a lot of advancement over the last decades. These are another class of molecular self-assembling systems that form nanostructures like strands, tapes, fibers, ribbons, platelets, and other aggregates with high aspect ratio upon gelation (Terech and Weiss, 1997). They form 3D network structures depending on their interaction with the solvent molecules, and disable the movement of organic solvents by capillary forces and surface tension. CPL materials can be fabricated by introducing a chiral source and achiral fluorophore which co-assembles through non-covalent interactions like hydrogen bonding, π-π stacking, and electrostatic interactions (Shen et al., 2015; Han et al., 2017). The intensity and handedness of CPL can be controlled by the controlling the interactions and position of achiral fluorophores on the template (Liu et al., 2018; Xing and Zhao, 2018). Herein, we describe the recent advancements in CPL systems created by the interaction of achiral dopants with chiral gelator.

Han et al. (2017) developed a CPL system by utilizing a *C*3 symmetrical L-glutamic acid-based host gelator 1,3,5-benzenetricarbonyl L-glutamate diethylester and AIE active dyes. The host gelator formed hexagonal nanotubes providing chiral space for encapsulating the AIE luminogen. The CPL signal showed a *g*_lum_ value in the order of 10^−3^ (Han et al., 2017). Another notable work, Li et al. (2019) demonstrated the development of CPL material containing chiral amphiphilic D-glutamic acid gelator (DGG) and achiral pyridine-functionalized tetraphenylethylenes (PTPE). An interesting feature of this work is the stoichiometry-controlled inversion of supramolecular chirality and CPL handedness. Left- and right-handed CPL were observed when the molar ratio of PTPE/DGG was 1:100 and 1:16, respectively (Li et al., 2019). Hydrogels have also been explored as a template to synthesize CPL materials. The CPL handedness of phenylalanine-based hydrogels was tailored by achiral isomers of naphthylamine through intermolecular hydrogen bonds and π-π stacking (Figure 2D). The CPL inversion is assisted by non-covalent interactions between the chiral and achiral molecules thereby inducing reorientations in the assembled molecules. The co-assembled hydrogels showed a |*g*_lum_| value in the order of 10^−3^ (Yang L. et al., 2019). Wang et al. (2019) chose chiral phenylalanine based hydrogelator in which achiral coumarin derivatives were co-assembled. The fabricated system exhibited high CPL performance with a *g*_lum_ value around −1.9 × 10^−2^. Even though the *g*_lum_ values are not very high, chiral gelators are emerging as promising templates for the development of a variety of CPL based composite systems.

## Polymers

The use of a chiral helical polymer templates for the generation of CPL is another area of attraction (Fukao and Fujiki, 2009; Nishikawa et al., 2017; Kulkarni et al., 2018). Zhao et al. (2018) reported a CPL material *via* co-polymerization of helical monosubstituted polyacetylene (PA) polymers and achiral fluorescent monomer in presence of a rhodium-based catalyst (Figure 2E). The achiral fluorescent monomers were attached to the polymer backbone through covalent bonds and CPL was generated only when helical structure of one handedness was formed (Zhao et al., 2018). They have further achieved a tunable CPL emission with high *g*_lum_ value of 0.1 using chiral helical substituted polyacetylenes (HSPAs) and fluorophores. The generation of CPL was attributed to the circularly polarized scattering and fluorescence-selective absorption effects of chiral HSPAs (Zhao et al., 2019). The CPL response from a syndiotactic polystyrene (s-PS) polymer films generated by the sorption of chiral molecules on crystallization was reported by Rizzo and coworkers. The polymeric film thus obtained, exhibited a CPL signal around 320 nm with a *g*_lum_ value of 0.03. CPL emission from an achiral fluorophore, fluorescein was also obtained on passing it through s-PS film (Rizzo et al., 2011, 2017).

## Other Templates

In addition to the templates described above, there are various other scaffolds employed for the fabrication of CPL active materials. Chiral silica is one such template that has been effectively utilized. Tsunega et al. (2019) have developed a CPL active material with inorganic silica as chiral host material with acidic fluorophores embedded in it. The surface modification of chiral silica provided the binding pocket for encapsulation of fluorophores. Recently, chiral nematic mesoporous silica films (CNMS) was used for generation of right-handed CPL from luminophores capped in it. Restricting the transmission of light with the same chirality, selective reflection band of CNMS enables R-CPL emission with *g*_lum_ value up to 0.38 (Jiang H. et al., 2019). In addition to these templates, chiral solvents have also been employed for generation for CPL in fluorescent materials and nanoparticles, however, we have restricted our discussions to template assisted chiral luminescence in organic fluorophores.

## Conclusions And Future Perspective

The major drawback associated with CPL studies on simple organic systems is the tedious protocols associated with the synthesis, purification and characterization of chiral molecules with high enantiopurity. Moreover, the systems exhibit low *g*_lum_ values. Molecular self-assembly has been demonstrated as an efficient technique to enhance the CPL of organic molecules. However, reproducibility and stability of the supramolecular systems is a major concern. The *g*_lum_ could be enhanced by an order of magnitude in most cases, which is still low for any technological applications. Under these circumstances, template assisted chiral induction has enormous potential and is gaining vast attention due to its attractive features. Template-driven CPL generation offers several advantages over the conventional CPL observed in molecular systems; (i) since the scaffold-based approach uses simple achiral fluorescent molecules, a wide range of molecules/dyes can be used, imposing minimal restrictions on the choice of molecular system, (ii) easy tuning of the emission wavelength is facilitated by the proper selection of molecules/dye, (iii) as highly ordered structures are used as templates, the chiral properties can be tailored through the control of template morphologies, (iv) the technique is cost effective as it can be applied on cheap host and guest materials, and (v) simple procedure involving mixing of the chiral host and achiral guest in most cases. While large number of templates have been employed, liquid crystalline materials have shown promise due to their unique selective reflection property. Since any chiral medium can function as a template for CPL generation, research is actively pursued to explore large number of novel templates capable of generating enhanced CPL. The incorporation of simple organic molecules into diverse chiral templates stipulates versatile strategy for the rational design and scalable manufacturing of organic-based CPL materials that can open new avenues for technological advances in field of photonic devices.

## Author Contributions

SM and JK conceived, designed the project, edited, and revised the review. SM, ABJ, and JK contributed to the writing and literature research. All the authors approved the final draft.

## Conflict of Interest

The authors declare that the research was conducted in the absence of any commercial or financial relationships that could be construed as a potential conflict of interest.
